# The alterations of the synthetic pathway and metabolic flux of auxin indole-3-acetic acid govern thermotolerance in *Lentinula edodes* mycelia subjected to heat stress

**DOI:** 10.1128/spectrum.01298-25

**Published:** 2025-11-28

**Authors:** Xiaoxue Wei, Jiaxin Song, Jiayue Chen, Yang Xiao, Yan Zhou, Yinbing Bian, Yuhua Gong

**Affiliations:** 1 Institute of Applied Mycology, College of Plant Science and Technology, Huazhong Agricultural University, Wuhan, Hubei, China; 2 Key Laboratory of Agro-Microbial Resource Comprehensive Utilization, Ministry of Agriculture, Huazhong Agricultural University, Wuhan, Hubei, China; Barnard College, New York, New York, USA

**Keywords:** *L. edodes*, integrated metabolome, transcriptome, IAA synthetic pathway, heat stress

## Abstract

Heat stress poses a significant constraint on the annual production of *Lentinula edodes (L. edodes*), a challenge that has been intensified by global warming. Previous studies have established a close relationship between intracellular indole-3-acetic acid (IAA) content and heat tolerance in *L. edodes*. However, the specific changes in the IAA synthesis pathway and the target genes regulated by IAA under heat stress remain unclear. We employed targeted metabolomics and transcriptomics analyses to investigate the alterations in IAA synthesis pathways and gene expression in both heat-tolerant and heat-sensitive strains at various time points during heat stress. Our findings revealed that IAA is primarily synthesized via the tryptamine (TAM) and indole-3-pyruvic acid (IPA) pathways in *L. edodes*. Heat-sensitive strain YS3357 exhibited excessive accumulation of TAM after heat stress. Silencing of the key genes of IAA synthesis, including tryptophan decarboxylase and tryptophan transaminase in strain S606, could reduce the thermotolerance of *L. edodes* mycelia. Transcriptome analysis revealed that heat-tolerant strain S606 had an earlier response to protein folding and mitochondrial gene expression compared to the heat-sensitive strain YS3357. Additionally, most genes in the mitogen-activated protein kinase signaling pathway were upregulated after 10–24 h of heat stress, with auxin response elements identified in their promoters. These results suggest that the excessive TAM accumulation is the newly discovered limiting factor for thermotolerance, and the expression levels of key genes in the IAA synthesis pathway could directly influence hyphal thermotolerance. This study provides a new perspective on the mechanism by which IAA and the synthesis precursors affect thermotolerance of *L. edodes*.

**IMPORTANCE:**

As an important plant hormone, the potential role of indole-3-acetic acid (IAA) in enhancing the heat resistance of *L. edodes* strains has garnered significant attention. This study systematically investigated the intracellular IAA biosynthesis pathway and its metabolic flows in *L. edodes* under varying durations of thermal stress, with particular emphasis on temporal gene expression patterns. Research has demonstrated that excessive accumulation of tryptamine may impair the heat stress recovery capability of *L. edodes*. In contrast, IAA can improve its thermotolerance by modulating the expression of genes associated with the mitogen-activated protein kinase signaling pathway.

## INTRODUCTION

Indole-3-acetic acid (IAA), a crucial phytohormone of the auxin class, plays pivotal roles in multiple aspects of plant growth and development. It actively participates in the regulation of embryo development (1), root organogenesis (2), environmental responses (3), adaptive growth (4) as well as cell division (5) and differentiation (1) processes. High-temperature-induced heat stress significantly impairs plant growth and development, ultimately leading to substantial reductions in crop yield and productivity.

Thermomorphogenic responses in plants involve coordinated regulation of auxin biosynthesis and transport following heat stress. Research across multiple species including soybean (6), *Arabidopsis* (7), lettuce (8), and cucumber (9) demonstrates significant auxin accumulation under thermal stress. The central thermomorphogenesis regulator, phytochrome-interacting factor 4 (PIF4) (10), orchestrates this process by upregulating key auxin biosynthesis genes (TAA1 family) while simultaneously modulating polar auxin transport through pin-formed 1 (PIN1) (11), and PIN2 (12) regulation. This molecular mechanism enables plants to optimize growth patterns and developmental plasticity under elevated temperature conditions.

Contemporary studies have demonstrated the regulatory potential of exogenous auxin application in plant physiology and stress responses. Notably, IAA supplementation has been shown to enhance the growth of soybean seedling (6), promote hypocotyl elongation in *Arabidopsis* (7), and mitigate heat-induced male sterility in wheat through improved grain retention (13), thereby reducing the loss of grain and increasing lettuce shoot (8). Furthermore, auxin application exhibits protective effects against thermal stress in rice cultivation, effectively maintaining pollen viability, spike fertility, and yield components under elevated temperatures (14).

Microbial-derived IAA has been documented to enhance plant resilience to various abiotic stresses, including saline, heavy metal (15), and drought stress (16). Recent investigations into thermal stress responses reveal that IAA production serves as a key adaptation mechanism in microorganisms, such as *Aspergillus japonicus* EuR-26 (17) and *L. edodes* (18). Low concentration of exogenous IAA increases the fungal growth, and at high concentration, it decreases the growth of the plant pathogen *Fusarium delphinoides* (19). Exogenous IAA suppressed *Magnaporthe oryzae* mycelial growth by inhibiting fungal endogenous IAA biosynthesis and delayed spore germination by impairing redox homeostasis (20). The phytopathogenic fungus *Leptosphaeria maculans* biosynthesizes auxins, and IAA production could be further stimulated by supplying precursors. Expression of indole-3-pyruvate decarboxylase *LmIPDC2*, tryptophan aminotransferase *LmTAM1,* and nitrilase *LmNIT1* genes was mainly upregulated after adding tryptophan and correlated with IAA production (21). Indole-3-acetic acid has also been detected in the model fungus *Saccharomyces cerevisiae*, and the aldehyde dehydrogenase involved in regulating IAA synthesis has been identified as a regulator of filamentation, with subsequent effects on virulence trait (22).

Specifically, our previous research identified a significant increase in endogenous IAA levels in *L. edodes* following a 24-h exposure to 40°C heat stress. Subsequent experiments demonstrated that exogenous application of IAA and its analogs (0.01 mmol/L 2,4-dichlorophenoxyacetic acid) significantly improved thermotolerance in selected *L. edodes* strains under identical thermal conditions (23). The above results indicate that the intracellular IAA content is closely related to hyphal heat tolerance, but the intracellular IAA synthesis pathway and the mechanism of IAA regulation of heat tolerance are still unclear. Current understanding of IAA biosynthesis reveals two primary routes in biological systems: tryptophan-dependent and tryptophan-independent pathways.

The tryptophan-dependent pathway involves L-tryptophan (Trp) conversion through five distinct enzymatic routes (24). The transformation process mainly consists of five pathways, namely IPA (indole-3-pyruvic acid), TAM (tryptamine), IAM (indole-3-acetamide), IAN (indole-3-acetonitrile), and TSO (tryptophan side-chain oxidase) pathways (18, 25–30). The IPA pathway, recognized as a principal auxin synthesis mechanism in microorganisms and plants, employs tryptophan aminotransferase (TAA/TAR) (31, 32) and flavin monooxygenase (YUCCA) (33) to convert Trp into IPA (34), subsequently transforming to IAA or via indole-3-acetaldehyde intermediates through pyruvate decarboxylase and indole-3-acetaldehyde (IAALD) dehydrogenase (ALDH) enzymes, as observed in *Ustilago maydis* (35) and *Tricholoma vaccinum-Spruce ectomycorrhiza* (36). The TAM pathway initiates with tryptophan decarboxylase (TDC) (37)-mediated formation of tryptamine, sequentially converted through N-hydroxy-tryptamine, indole-3-acetaldoxime (IAAOx), and IAALD to final IAA production (38), with functional validation in *Taphrina deformans* (39). The IAM pathway utilizes the IaaM (encoding tryptophan monooxygenase) and IaaH (encoding indole-3-acetamide hydrolase) (40) to convert tryptophan to IAM and subsequently to IAA, originally identified in *Pseudomonium* (41) and later confirmed in *Bradyrhizobium japonicum* (42), *Rhizobium* sp. strain NGR234 (43), and *Burkholderia pyrrocinia* JK-SH007 (44). The IAN pathway, predominantly studied in bacteria, involves the conversion of Trp to IAAOx by cytochrome P450 monooxygenases (CYP79B1, B2, B3) (45) followed by transformation into IAN and subsequent hydrolysis to IAA by nitrilase encoded by the *NIT* (encoding nitrilase) gene. This pathway has been identified in *P. fluorescens* EBC191 and *Alcaligenes faecalis* JM3 (46–48). The TSO pathway has only been shown in *Pseudomonas fluorescens*, and in this pathway, Trp is directly converted to IAALD by passing IPA (28). Comparatively, research on the tryptophan-independent pathway remains limited, though elevated IAA levels detected in *Arabidopsis* tryptophan biosynthesis mutants suggest the existence of alternative synthesis routes (49).

Given the critical role of auxin in plant growth and development, with key biosynthesis genes including *TDC* and *TAA. TDC* gene mediates Trp-to-TAM conversion, with overexpression studies demonstrating enhanced cell elongation in tobacco BY 2 cells (50), improved drought and salt stress tolerance in *Paeonia lactiflora Pall* (51)*,* promoted the root length, root surface area, and leaf thickness of tomato (52), delayed senescence, and improved resistance to pathogen infection in rice (53).

*TAA* gene catalyzes the critical Trp-to-IPA conversion in auxin biosynthesis, expression modulated by environmental stimuli such as shade avoidance responses to strong light stress (54). The expression of the *TAA* gene is regulated by environmental factors such as temperature signaling (55). Genetic studies in deletion mutants of *TAA* family alleles (*sav3*, *wei8*, *tir2*) (21, 56) reveal its pleiotropic effects on plant development, including altered tiller number but reduction in grain number and size (57), exhibit a defective root gravitropic response and an increased resistance to cytokinin (CK) in primary root growth (58) as well as auxin content (59). Exogenous addition of IAA can complement these phenotypic defects.

Despite these advances in plant and bacterial research, significant research gaps persist regarding IAA biosynthetic pathways and their regulatory roles in edible fungi, and current limitations include incomplete characterization of fungal-specific IAA synthesis genes, undefined IAA synthesis pathway predominance under thermal stress, and insufficient understanding of cross-talk between auxin signaling and heat response pathways in *L. edodes. L. edodes* have emerged as a significant horticultural crop owing to their distinctive flavor and high nutritional value (60). High temperature has a great negative influence on the cultivation of *L. edodes*, causing mycelium damage and stem rot, thus affecting yield, usually resulting in a reduction of 30% (61). In previous studies, *L. edodes* regulated hyphal thermotolerance by regulating intracellular IAA content, but the intracellular IAA synthesis pathway and the mechanism of IAA regulating thermotolerance are unknown. This study analyzed the dynamic changes of plant hormone and gene expression after 40°C heat stress at 2, 4, 6, 12, and 24 h by quantitative metabolome and transcriptome methods. This study systematically investigated the intracellular IAA biosynthesis pathway and its metabolic flows in *L. edodes* under varying durations of thermal stress, with particular emphasis on temporal gene expression patterns. Through targeted silencing of two pivotal biosynthetic genes tryptophan aminotransferase and tryptophan decarboxylase, we elucidated their regulatory roles in hyphal thermotolerance. This investigation not only elucidates the mechanistic basis by which auxin modulates thermotolerance in *L. edodes* hyphae under heat stress but also establishes a novel insight for understanding how auxin homeostasis coordinates heat stress adaptations through metabolic flux regulation and differential gene transcriptional expression within the IAA biosynthesis network.

## MATERIALS AND METHODS

### Strains and culture conditions

*L. edodes* YS3357 and S606 strains used in this study were supplied and preserved exclusively by the Institute of Applied Fungi Research, Huazhong Agricultural University. *L. edodes* mycelia were cultivated on malt yeast extract glucose (MYG) medium (containing 20 g malt extract, 20 g glucose, 1 g tryptone, 1 g yeast extract, 20 g agar, 1 L ddH_2_O) in 9 mm petri dish at 25°C for 8 days. In this study, for metabolomic analysis, samples of the two strains were collected after cultivation at 25°C for 8 days, samples were harvested before heat shock treatment at 40°C (CK), as well as at 6, 12, and 24 post-treatment. For transcriptomic analysis, samples of the two strains were collected following 8 days of cultivation at 25°C. Sampling time points comprised the pretreatment control (CK) and 2, 4, 6, 12, and 24 h after exposure to 40°C heat shock (62).

### Quantitative analysis of plant hormones and related metabolites

The *L. edodes* strains YS3357 and S606 subjected to 40°C heat stress for different durations were used for metabolite extraction. Extraction and determination of metabolites were performed by Wuhan Metware Biotechnology Co. Ltd. A total of 0.1 g hypha samples were extracted with 1 mL of 70% methanol solution at 4°C for 24 h. The extract was vortexed for 10 min and centrifuged for 5 min (12,000 rev/min, and 4°C), and the supernatant was then transferred to clean plastic microtubes and allowed to air-dry. The dried samples were dissolved in 100 µL 80% methanol (vol/vol) and filtered through a 0.22 µm membrane filter for further LC-MS/MS analysis (63). Metabolites were detected using ultraperformance liquid chromatography (Shim-pack1 https://www.metware.cn/, UFLC SHIMADZU CBM30A) and tandem mass spectrometry (Applied Biosystems 6500 QTRAP, Wuhan, China). The column temperature was 40°C, the flow rate was 0.4 mL·min^−1,^ and the injection volume was 2 µL. Mobile phase A was 0.04% acetic acid aqueous solution, and mobile phase B was acetonitrile containing 0.04% acetic acid. The gradient elution was carried out with elution conditions presented in Table S1. The voltage of the mass spectrometer was 5,500 V, the temperature of the electrospray ion source was 500°C, and the curtain gas was 25 psi. Qualitative analysis of samples was performed according to the secondary spectrum information in the Metware Database. The relative content of metabolites was calculated according to the corrected mass spectrum peak area. The differentially accumulated metabolites (DAMs) between different groups were identified with the thresholds of |log_2_ FC (fold change)| ≥2 and *P* < 0.05.

Indole-3-acetaldehyde, an intermediate metabolite in IAA synthesis, was tested by using an ELISA kit (Shanghai Hengyuan Biotechnology Co., Ltd., Shanghai, China). Briefly, 50 µL samples were added to the standard on the coated plate, and 10 µL of the sample dilution (5× final dilution of the sample) was added to the 96-well plate, added with 50 µL of enzyme labeling reagent (except for blank wells), then gently shaken to mix evenly. The microplate was covered with plate sealing film and incubated at 37°C for 60 min. The 30× concentrated washing solution was diluted 30-fold with distilled water. The plate sealing film was carefully removed, and the liquid was discarded. After spin drying the samples, the washing solution was added to each well. Following a 30-s incubation to allow precipitation, the supernatant was discarded. The above procedures were repeated five times, and then the samples were spin dried. The dried samples were added with 50 µL color development reagent A, then added with 50 µL of color development reagent B, gently shaken, mixed at 37°C, followed by a 15-min color development, and finally added with 50 µL of stop solution to terminate the reaction (blue turning yellow). The absorbance (indicated by optical density, OD) at 450 nm was measured within 15 min after the addition of stop solution. Afterward, the standard curve was plotted with the concentration of the standard as the abscissa and the OD value as the ordinate. Then the actual sample concentration was calculated according to the OD value and standard curve.

### Total RNA extraction and reverse transcription

The 0.1 g of *L. edodes* fresh hypha was taken, added with liquid nitrogen, quickly ground into powder, transferred to RNase-free 1.5 mL centrifuge tube, added with 600 µL RNAiso Plus, mixed evenly, the mixture was incubated at room temperature for 5 min, followed by the addition of 0.2 volumes of chloroform. After vortexing for an additional 20 s, the sample was incubated again at room temperature for 5 min and then centrifuged at 13,000 g for 10 min. The supernatant was transferred to a new RNase-free 1.5 mL microcentrifuge tube, mixed with 2/3 volumes of isopropyl alcohol, incubated at room temperature for 10 min, and centrifuged at 13,000 × *g* for 10 min at 4°C. The supernatant was discarded, and the RNA pellet was washed twice with 1 mL of 70% ethanol, with residual ethanol removed by brief centrifugation and aspiration. The pellet was air dried on an ultra-clean workbench for 5 min, resuspended in 30 µL of RNase-free ddH_2_O by incubating at 65°C for 5 min, and immediately stored at −80°C for subsequent transcriptome sequencing. The sample concentration was determined using an ultra-trace UV spectrophotometer, and the RNA integrity was measured by 1% agarose gel electrophoresis. The reverse transcription of RNA was performed using the HiScript II Q RT SuperMix Kit (Vazyme, Nanjing, China) at 42°C for 2 min in 8 µL reaction system containing 400 ng total RNA, 2 µL 4× gDNA wiper mix, and RNase-free ddH_2_O. In order to achieve high reverse transcription efficiency, the template was mixed with the RNase-free ddH_2_O. The mixture was homogenized and incubated at 65°C for 5 min, then added with another 4× gDNA wiper Mix to remove gDNA, added with 2 µL 5 HiScript II qRT SuperMix II, and gently mixed evenly. The subsequent reverse transcription procedures were as follows: at 25°C for 10 min, 55°C for 30 min, and 85°C for 5 s. The reverse transcription product was 20-fold diluted and used for later qRT-PCR analysis.

### Identification of differentially expressed genes and cluster analysis

The samples used for transcriptome analysis were collected by the same method as mentioned above for the metabolomics analysis in section quantitative analysis of plant hormones and related metabolites. After RNA extraction, the library was constructed. Sequencing was carried out using the Illumina HiSeq platform. After the quality control of the library, 150 bp paired-end clean reads were obtained. The sequence alignment was conducted with the L808 genome as the reference sequence (64). The genes were counted using Feature Counts software, and the gene expression level was expressed as fragments per kilobase of transcript per million mapped reads. The differentially expressed genes (DEGs) between comparison groups were identified using DESeq2 software (60), with the thresholds of |log2 FC| ≥2 or FDR (false discovery rate) <0.05. The Benjamin-Hochberg method was used to correct *P*-values and to obtain the false discovery rate. Kyoto Encyclopedia of Genes and Genomes (KEGG) analysis was conducted on the DEGs identified using BLAST software. To verify the expression of DEGs, the qRT-PCR was used. Specific quantitative primers were designed using Primer 6.0 software (Table S2). The quantitative analysis was performed using Tip Green qPCR SuperMix, and the reaction system contained the upstream and downstream fluorescent quantitative primers (1 µL), TransStart Tip Green qPCR SuperMix (10 µL), cDNA (2 µL), and ddH_2_O (6 µL). The qRT-PCR reaction procedures were shown in Table S3. Gene expression levels were determined by the 2^−ΔΔCT^ method, with *Leactin* as the internal reference gene (65).

### Plasmid construction and fungal transformation

The modified pCAMBIA1300-g vector containing the tryptophan decarboxylase and tryptophan aminotransferase genes was used to construct the RNAi plasmids of *LeTAA* and *LeTDC* genes (66). The promoter of *Legpd* (glyceraldehyde-3-phosphate dehydrogenase) and terminator of T-nos were used to control the expression of *LeTAA* and *LeTDC* and their sense and antisense sequences (65). The RNAi vector was constructed, as described in a previous study. The 500 bp antisense fragments on conserved domains of *LeTAA* and *LeTDC* genes were PCR amplified, with the cDNA of S606 as a template. The *Leactin* promoter, antisense fragment, *Legpd* promoter, and the linearized pCAMBIA1300-g plasmid were linked together by homologous recombination to generate *LeTAA-*RNAi (Fig. S1A) and *LeTDC*-RNAi vectors (Fig. S1B) (67). *LeTAA-*RNAi and *LeTDC*-RNAi were transformed into strain S606 by *Agrobacterium tumefaciens*-mediated transformation method (67). The empty vector PCAMBIA1300-g was transformed into S606 as control (CK). MYG medium supplemented with 6 µg/mL hygromycin B was used to select positive transformants. Whether the transformants were successfully transformed with *TAA or TDC* fragment was verified by PCR.

### Statistical analysis

SPSS 19.0 (IBM Corporation, United States) was used to determine significant differences between groups and conduct one-way ANOVA and Duncan multiple range tests. *P* < 0.05 was considered statistically significant. GraphPad Prism software was used for graphing. Subsequently, the normality of the data was assessed using the one-sample Kolmogorov-Smirnov test. The data were presented as mean ± SD. Non-normal variables were reported as median (interquartile range). Means of two continuous normally distributed variables were compared by independent samples Student’ *t* test. Mann-Whitney *U* test and Kruskal-Wallis test were employed to compare non-normally distributed data from multiple groups, respectively.

## RESULTS

### Higher heat tolerance to 40°C heat stress of heat-tolerant strain than heat-sensitive one

**Fig 1:**
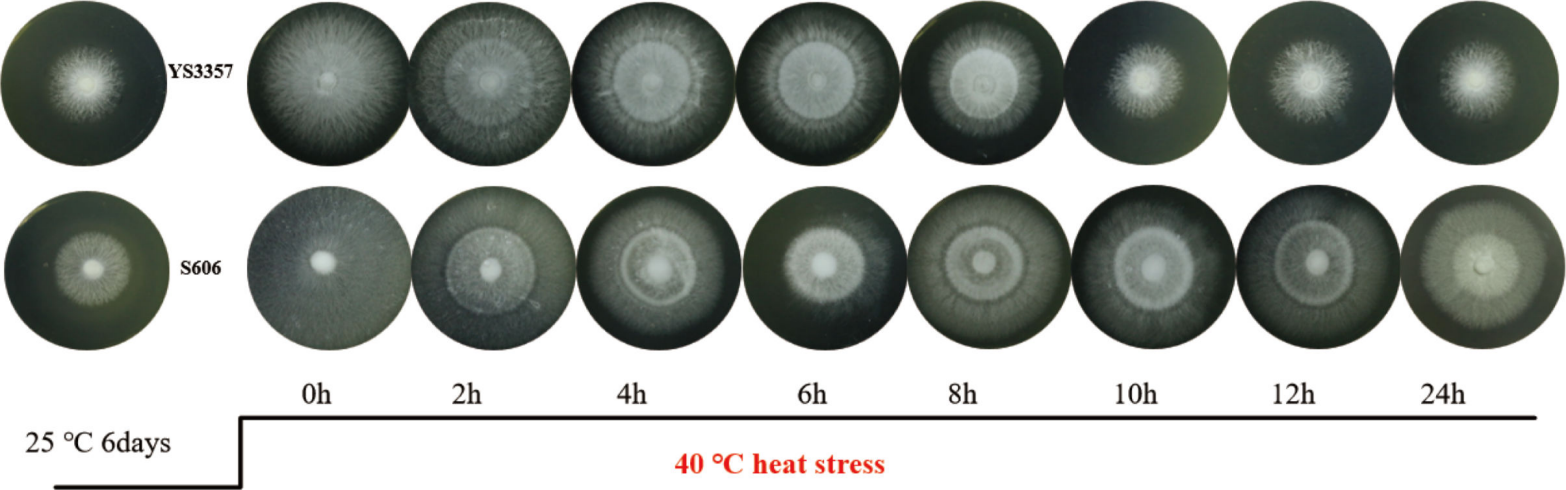
Hyphal growth dynamics under heat stress and recovery. *L. edodes* strains S606 (heat tolerant) and YS3357 (heat sensitive) were cultured at 25°C for 6 days, subjected to 40°C heat stress for indicated durations (0–24 h), and allowed to recover for 7 days. Images depict hyphal morphology post-recovery.

In this study, we established a model to compare the heat tolerance of various *L. edodes* strains. After 40°C heat stress for 2, 4, 6, 8, 10, 12, and 24 h, the mycelial growth recovery ability of *L. edodes* heat-sensitive strain YS3357 and heat-tolerant strain S606 at 24°C post 7-day culture was investigated (Fig. 1). After 10–24 h of 40°C heat stress, the strain YS3357 failed to resume growth, while strain S606 resumed growth at all the test time points, indicating that 10 h of 40°C heat stress is a key point for distinguishing ability of thermotolerance on different *L. edodes* strains.

### Target metabolomic analysis of phytohormones and related metabolites under different durations of heat stress

To deepen our understanding of the correlation between phytohormone and thermotolerance of *L. edodes* strains, we performed targeted metabolomic analyses of nine types of main phytohormones and their related metabolites in *L. edodes* strains S606 and YS3357 hypha after different durations of heat stress. Seven phytohormones, including cytokinin, indole-3-acetic acid, jasmonic acids (JA), gibberellin acids, abscisic acids, salicylic acid (SA), and ethylene, as well as 43 phytohormone-related metabolites from *L. edodes* mycelia were successfully detected (Table S4). Two target phytohormones, melatonin and (±) strigolactones, were undetected from *L. edodes* mycelia.

**Fig 2:**
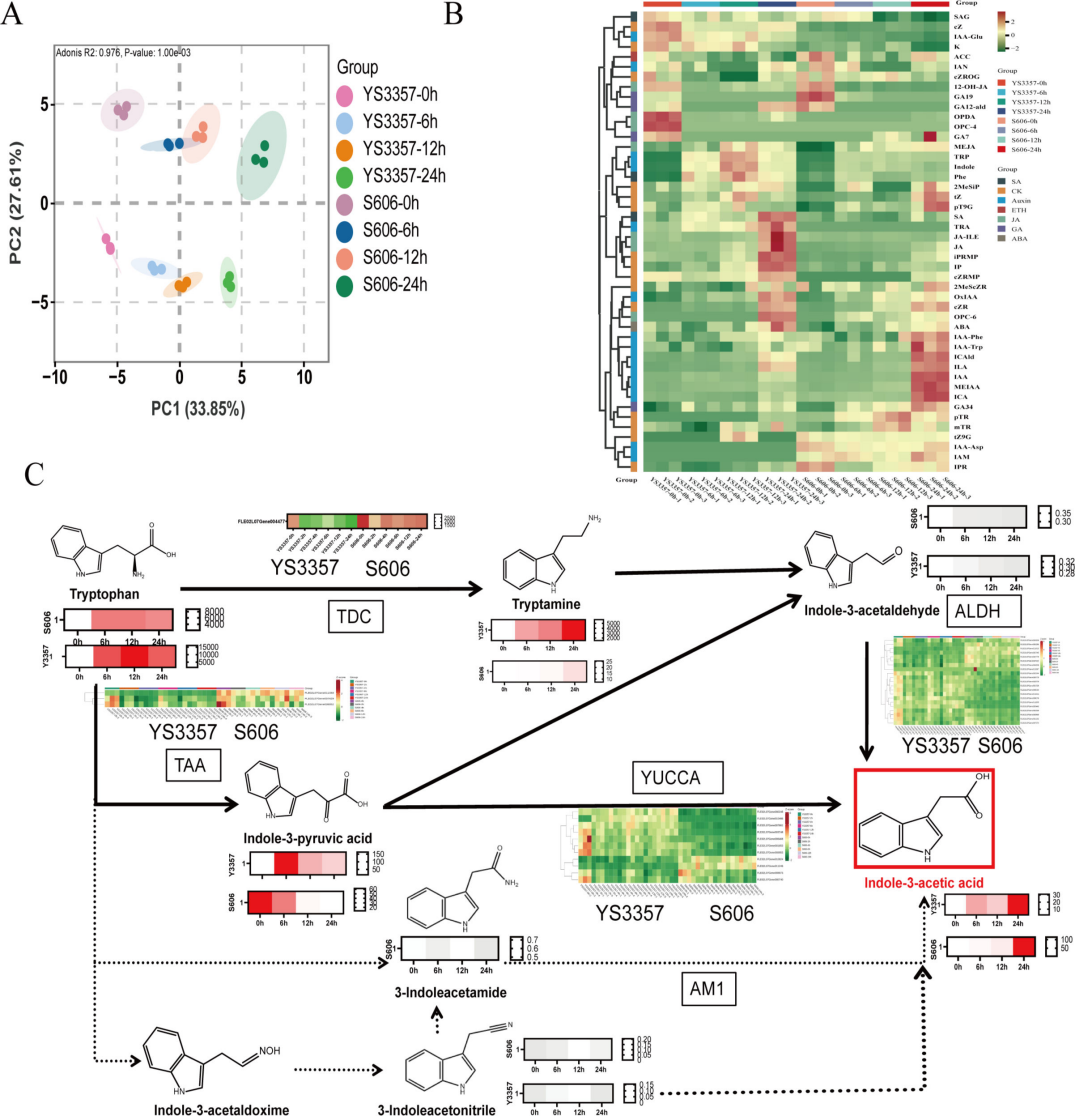
Metabolic profiling of fungal strains during heat stress. (**A**) Principal component analysis of differentially accumulated metabolites at 0, 6, 12, and 24 h of 40°C stress. (**B**) Heatmap clustering of DAMs across time points. (**C**) Indole-3-acetic acid metabolic flux in S606 vs YS3357 under stress. Color intensity corresponds to metabolite abundance (darker = higher).

Principal component analysis (PCA) of normalized metabolite expression levels and orthogonal projections to latent structures analysis differentiated the thermotolerance of *L. edodes* strains S606 and YS3357 along the second principal component (PC2) (Fig. 2A). The content of IAA increment was greater in heat-tolerant strain S606 than in heat-sensitive strain YS3357, with S606 rising from 2.67 to 113.77 ng/g, YS3357 going up from 1.94 to 30.88 ng/g, while the contents of SA and JA were increased only in strain YS3357. The analysis of target metabolomics indicated that the heat tolerance of hyphal to 24 h heat stress was highly correlated with the intracellular IAA content, and the accumulation of other hormones could not enhance the heat tolerance of *L. edodes* strains (Fig. 2B).

**Fig 3:**
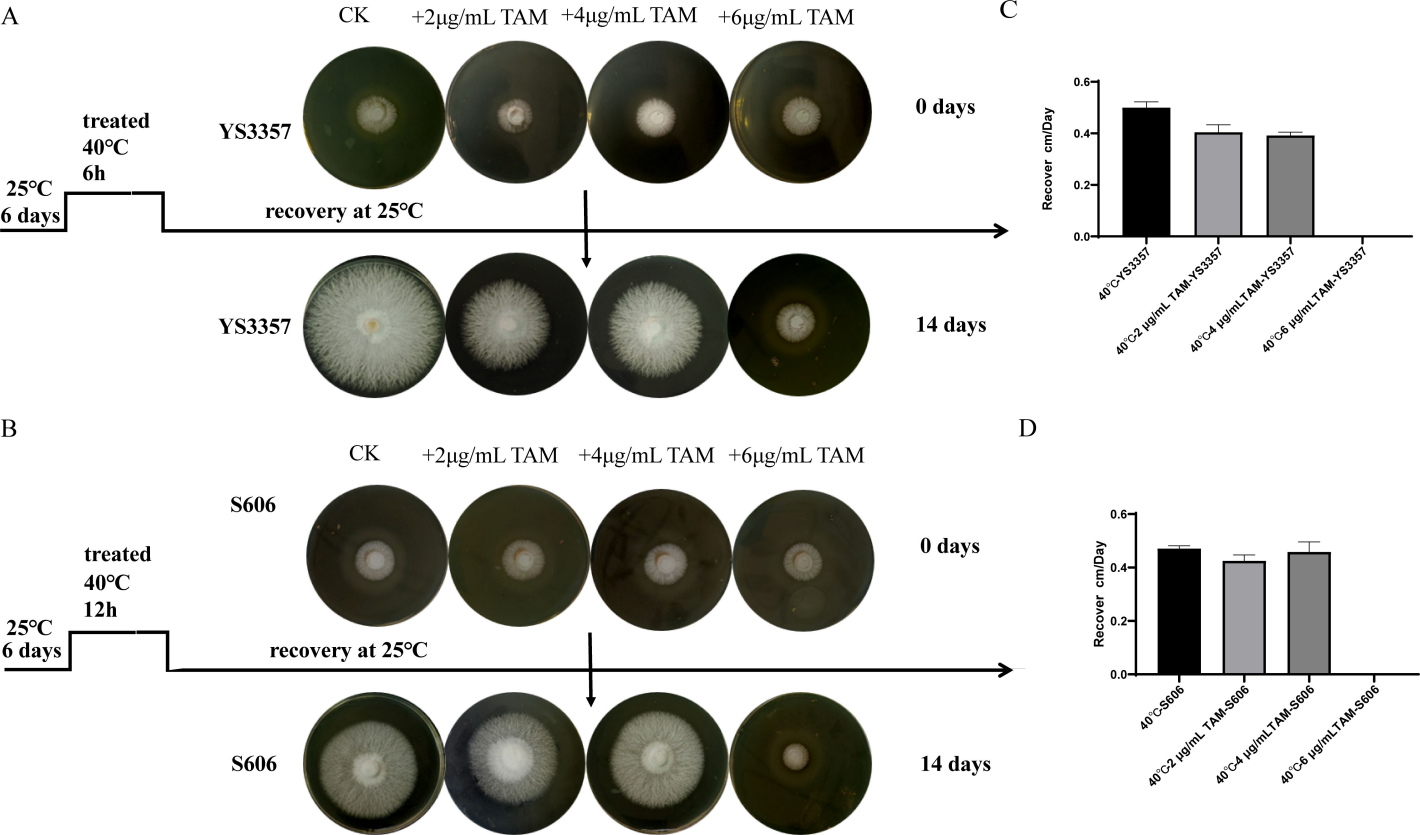
Phenotypic recovery of mycelial growth post-heat stress with exogenous tryptRecovery of Mycelial Growth Post-Heat Stress with Exogenous Tryptamine. (**A**) Heat-sensitive strain YS3357 recovery (40°C, 6 h), (**B**) hHeat-tolerant strain S606 recovery (40°C, 12 h), (**C**) grGrowth rate recovery: hHeat-sensitive strain YS3357, and (**D**) grGrowth rate recovery: h Heat-tolerant strain S606.

Analysis of IAA synthesis intermediates following heat shock revealed that changes in IPA and TAM are linked to the heat stress response in two strains (Fig. 2C). Detailed content data are provided in Table S4. Notably, excessive accumulation of TAM was observed in the heat-sensitive strain YS3357. In this study, *L. edodes* mycelia were cultured at 25°C for 8 days. The average tryptamine content in the heat-tolerant strain S606 was 7.44 ng/g, significantly lower than the 1426.90 ng/g observed in the heat-sensitive strain YS3357, representing a difference of approximately 200-fold. After 24 h of heat stress, TAM levels increased to 25.43 ng/g in the heat-tolerant strain and rose dramatically to 6262.77 ng/g in the heat-sensitive strain, resulting in a roughly 250-fold difference. These increases corresponded to 241.86% and 338.89% in heat-tolerant and heat-sensitive strains, respectively. Notably, the heat-sensitive strain YS3357 exhibited exceptionally high TAM accumulation. Given the substantial intracellular TAM levels observed in YS3357 after heat stress (6262.77 ng/g), we sought to investigate the underlying mechanisms by exogenously applying TAM to both the heat-tolerant strain S606 and the heat-sensitive strain YS3357. TAM was dissolved in dimethyl sulfoxide, and three final concentrations were tested in the growth medium: 2,000, 6,000 (selected to approximate the endogenous level in YS3357 post-stress), and 12,000 ng/g. The effects of these TAM treatments on mycelial growth and heat resistance phenotypes were subsequently evaluated. Following heat shock at 40°C, the recovery phenotypes of the treated strains were assessed. The results are presented in Fig. 3. Exogenous tryptamine application at all tested concentrations did not affect mycelial growth (Fig. S4). However, high-concentration tryptamine treatment significantly reduced the recovery capacity of *L. edodes* mycelia following heat stress at 40°C (Fig. 3A and B); it had no significant effect on the growth rate after recovery from heat stress (Fig. 3C and D).

Building on this finding, we further explored whether the expression trends of auxin biosynthetic genes are consistent with the variation of key intermediate metabolites TAM and IPA. As shown in Fig. S5; Fig. 2C, both transcriptome and qPCR analyses demonstrated that the expression level of the *LeTAA* gene was downregulated in both strains. In strain S606, this downregulation was consistent with the decrease in IPA content; however, in strain YS3357, it was inconsistent with the observed reduction in IPA. Transcriptomic data further revealed that the expression of the *LeTDC* gene was downregulated in both strains, which contradicted the observed increase in TAM content. In contrast, qPCR results revealed distinct expression patterns of the *LeTDC* gene at multiple heat shock time points, differing from the transcriptome findings. Specifically, in the heat-tolerant strain, *LeTDC* expression increased after 4 h of heat shock, while in the heat-sensitive strain, it was upregulated at 2, 6, 10, and 24 h post-heat shock trends that aligned with the increase in TAM levels.

### Silencing of key genes *LeTAA* and *LeTDC* in the IAA synthesis pathway can affect the heat tolerance of *L. edodes* strains.

**Fig 4:**
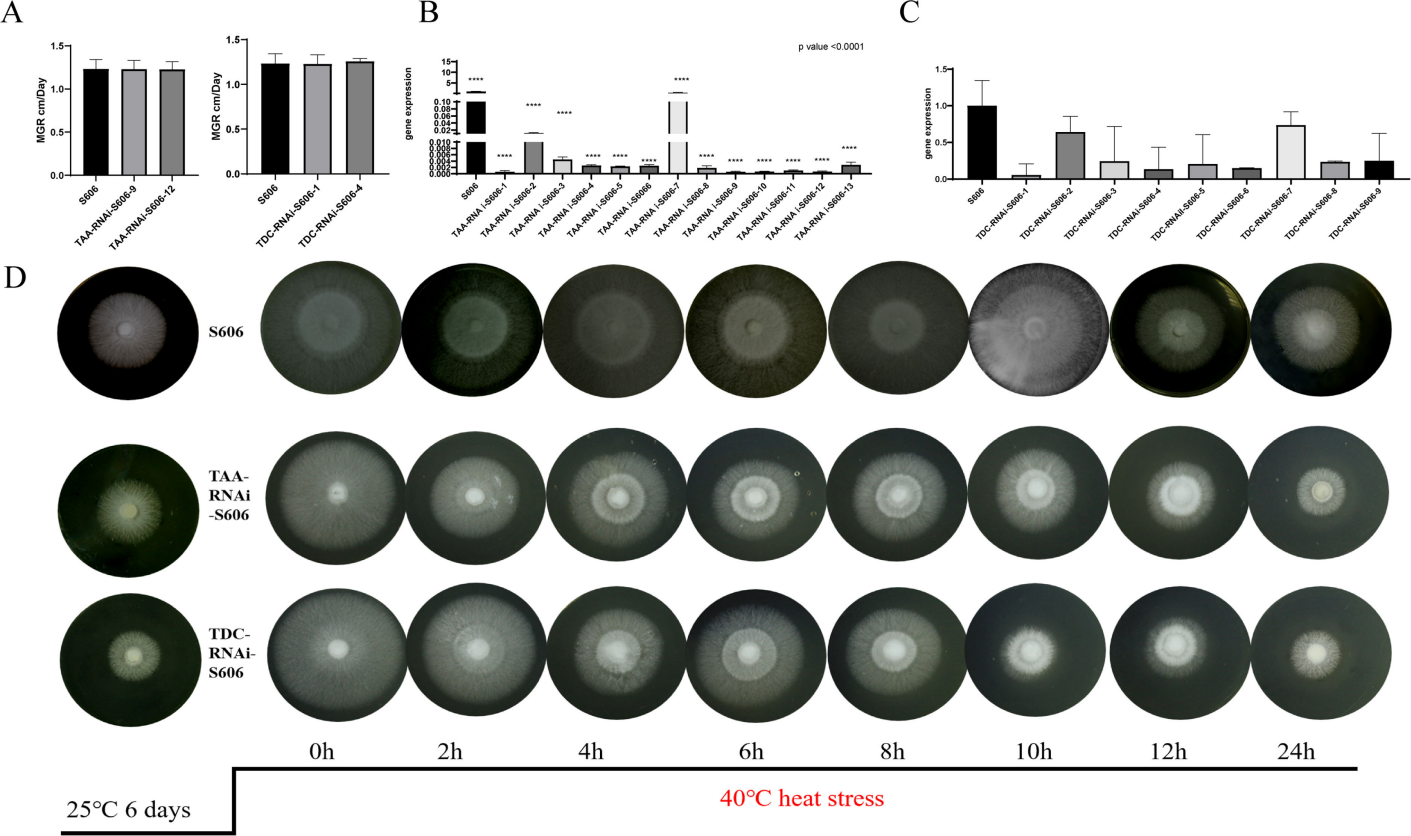
Functional validation of heat tsolerance-associated genes. (**A**) Growth rates of S606 and transformants (TAA-RNAi and TDC-RNAi). (**B and C**) Relative expression of *LeTAA* and *LeTDC* in S606 vs transformants (****P < 0.0001, *t*-test). (**D**) Recovery phenotypes after 40°C shock. Data represent mean ± SD (*n* = 6).

According to the above findings, *L. edodes* strains mainly produce IAA via the IPA and TAM pathways. To further reveal the IAA synthesis mechanism under heat stress, we investigated the key gene *LeTAA* and *LeTDC* functions in IPA and TAM pathways. Nine positive transformants each of the *LeTAA* and *LeTDC* genes were obtained based on the two-promoter silencing system, compared with those in wild-type strains S606 and YS3357, and the expression levels of *LeTAA* and *LeTDC* genes in transformant-containing strains were decreased by approximately 50%. Three of the transformants (TAA-RNAi-YS3357-12, TAA-RNAi-S606-13, and TDC-RNAi-S606-9) were selected with wild-type S606 for subsequent analyses. The results showed that there were no significant differences in mycelial growth rate and colony morphology on MYG medium between three transformant strains and two wild-type strains (S606) (*P* > 0.05) (Fig. 4A). These results suggested that the interference with *LeTAA* and *LeTDC* genes had no significant influence on *L. edodes* strain growth. At 24 h post 40°C heat stress, TAA-RNAi-S606-13 and TDC-RNAi-S606-9 transformant strains could not resume growth. At 12 h, transformant strains resumed growth, but their recovery phenotype was inferior to that of the wild-type S606, indicating the interference of *LeTAA* and *LeTDC* genes in heat-tolerant strains diminished their heat resistance (Fig. 4B and C). Since it mainly synthesized IAA through the TAM and IPA pathways, the interference of the genes of TAA and TDC might have attenuated the heat tolerance of the mushroom by affecting the synthesis of IAA (Fig. 4D).

### Analysis of gene expression changes during 0–24 h of 40°C heat stress in *L. edodes* strains

**Fig 5:**
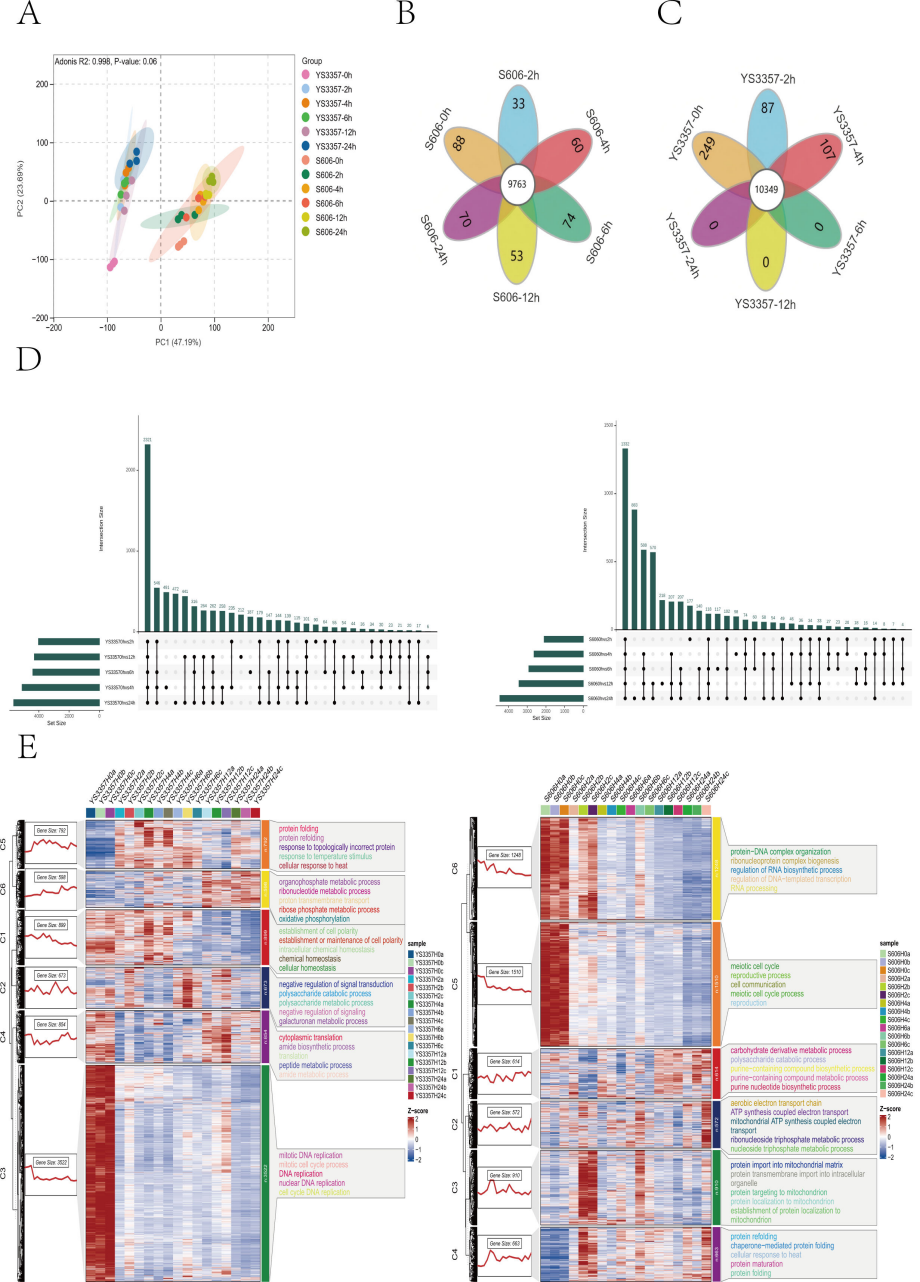
Transcriptomic analysis of heat stress responses. (**A**) PCA of global gene expression. (**B and C**) Venn diagram of strain-specific genes. (**D**) Differentially expressed genes (DEGs) at each stress time point in S606 and YS3357 strains. (**E**) Expression trends of heat-responsive genes and enriched KEGG pathways in S606 and YS3357 strains. (FDR < 0.05).

We further performed a transcriptomic analysis of the expression of the two strain genes during 0–24 h (0, 2, 4, 6, 12, and 24 h of heat stress at 40°C). PCA analysis showed that the gene expression patterns of S606 and YS3357 strains were clearly separated in the second component and then in the first component after heat stress (Fig. 5A). It can be seen in the Upset plot that the number changes of DEGs in the two strains showed different patterns at different times of heat stress, with the heat-resistant strain S606 increasing with increasing heat stress time, peaking of 4,582 genes at 24 h. The heat-sensitive strain YS3357 produced a high number of differential genes, i.e., 4,111, after 2 h of heat stress (1.5 times that of S606) and aAt a peak of 5,817 genes after 24 h of heat shock, indicating that gene expression is unstable under YS3357 heat stress (Fig. 5D and E). In heat-tolerant strain S606, 1,486 genes were upregulated after 2 h of heat stress (clusters 3 and 4), which were mainly related to protein-related mitochondrial localization and target to the mitochondria, protein folding, protein refolding, cell response to heat, and temperature stimulation pathways. However, the gene expression pattern of heat-sensitive strain YS3357 in response to heat stress was quite different from that of S606. About 50% of the genes were significantly downregulated after 2 h of heat stress, and only 792 genes (cluster 5) were upregulated after 2 h of heat stress, and the upregulated genes were mainly related to protein folding and refolding pathways. These results indicate that the heat-resistant strain S606 gained thermostability by the earlier upregulated gene expression strategy (Fig. 5F and G).

### *L. edodes* strains exhibited enhanced heat tolerance through IAA-mediated regulation of the MAPK signal transduction pathway during 40°C heat stress at 0–24 h

**Fig 6:**
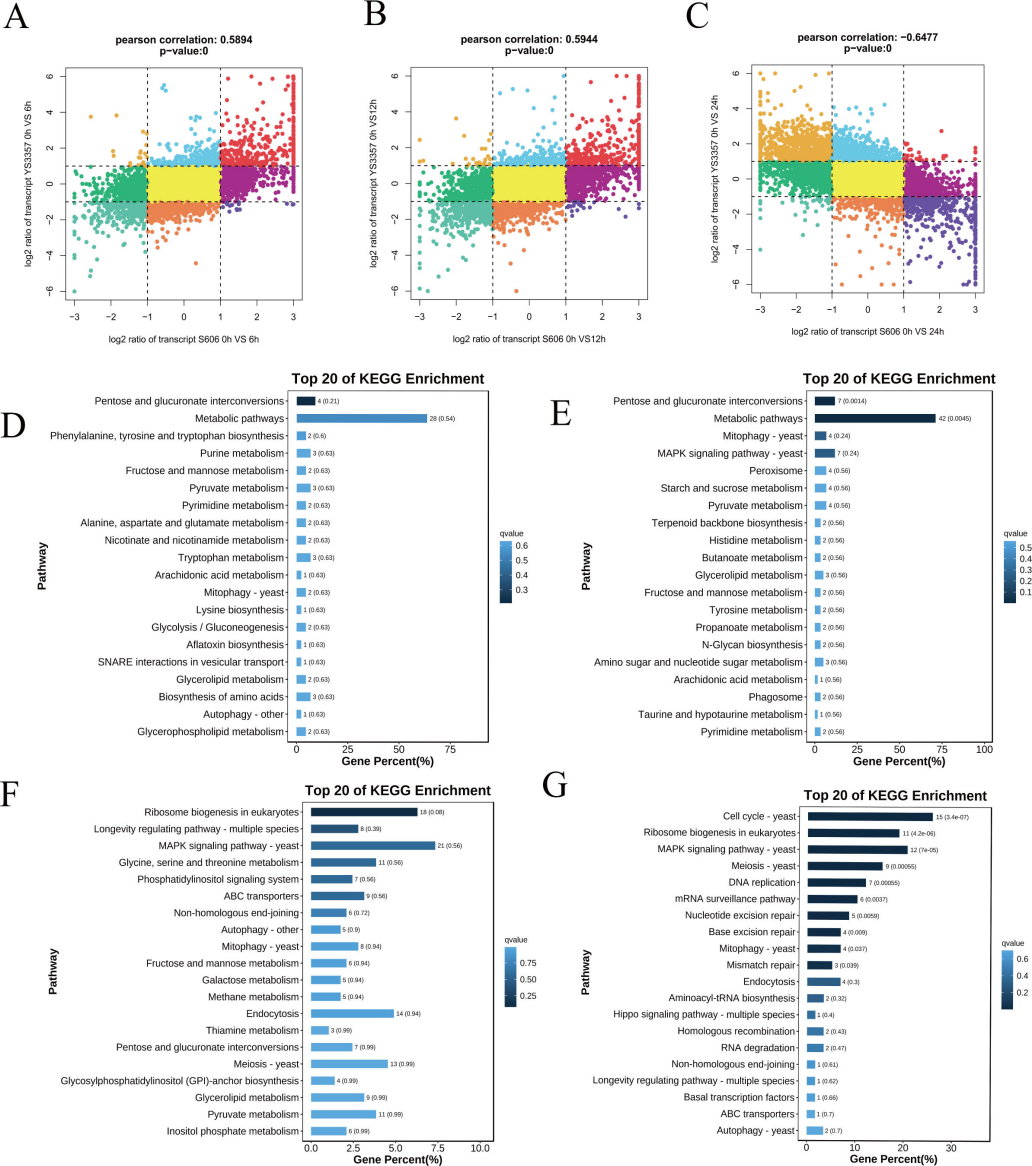
Mechanistic dissection of thermotolerance divergence between *L. edodes* strains YS3357 and S606 using nine-quadrant clustering and OP2LS analysis. (A–C) Dynamic differential gene expression patterns in the heat-tolerant strain S606 during 40°C heat shock at 6 h (**A**), 12 h (**B**), and 24 h (**C**). Clusters were identified via nine-quadrant strategy (|log2FC| >1, FDR <0.05). (D–G) Time-resolved KEGG pathway enrichment of heat shock-responsive differentially expressed genes at 0 h (**D**), 6 h (**E**), 12 h (**F**), and 24 h (**G**). Top enriched pathways (FDR < 0.05) are labeled.

**Fig 7:**
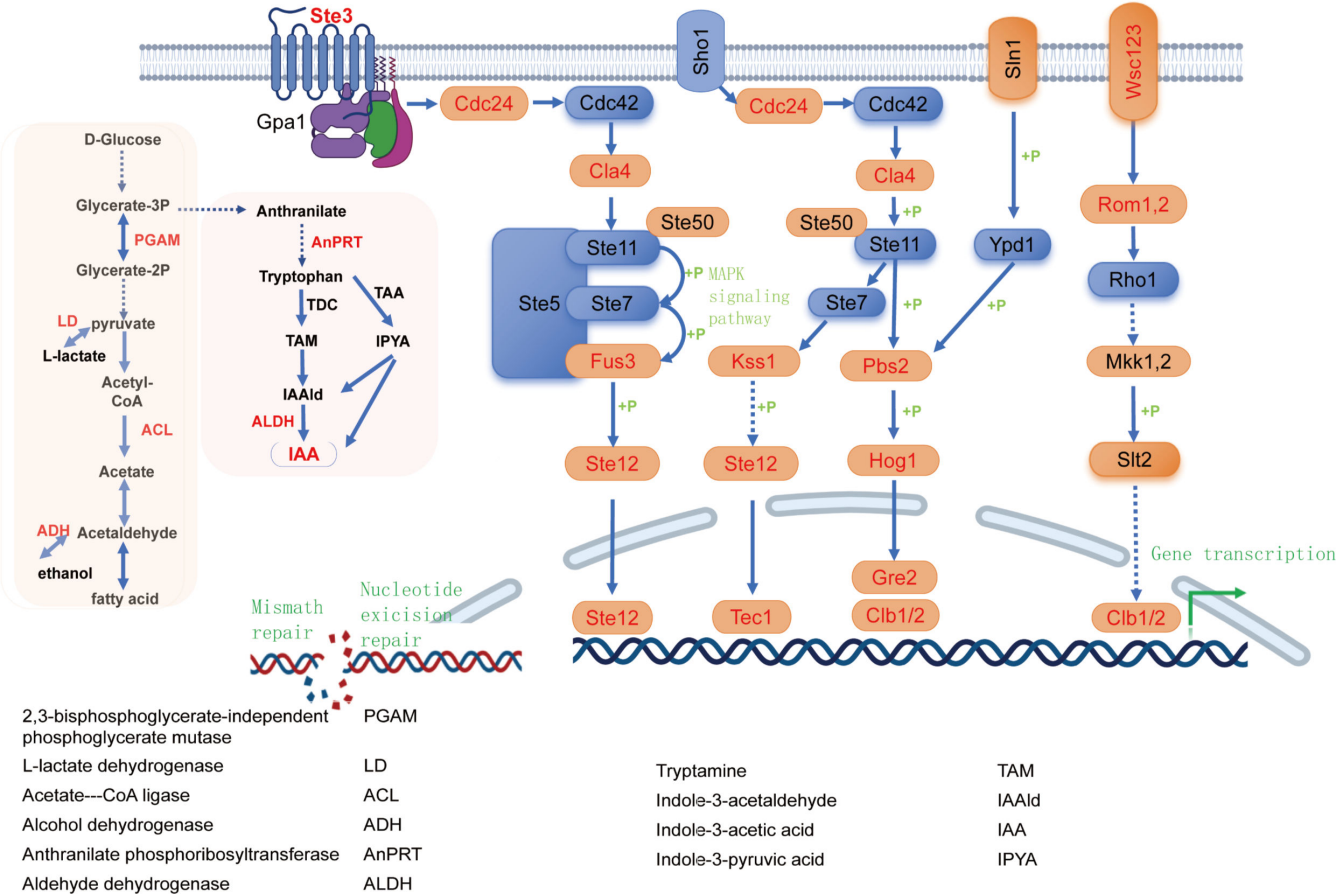
Proposed model of IAA-mediated MAPK pathway regulation under heat stress. Red-labeled pathways (fatty acid metabolism and IAA synthesis) indicate upregulated genes. Auxin response factor-responsive elements (red) in MAPK components suggest auxin crosstalk.

To investigate the mechanisms underlying the remarkable heat tolerance of the S606 strain during heat stress, this study employed a nine-quadrant analysis to compare the DEGs (Tables S5 and S6) between S606 and YS3357 at 0–24 h post-heat exposure (Fig. 6A through C). The analysis indicated that genes situated in the first and second quadrants, which were highly expressed in S606 but either downregulated or unchanged in YS3357 following heat stress, were closely associated with the superior heat tolerance observed in S606. Notably, after 6, 12, and 24 h of heat shock, the number of genes in these quadrants increased, suggesting that the S606 strain effectively mitigated heat stress by upregulating a greater number of genes compared to the YS3357 strain. KEGG enrichment analysis of gene functions in the first and second quadrants demonstrated distinct functional changes in high DEGs in the S606 strain during 0–24 h of heat stress. KEGG enrichment analysis of gene functions in the first and second quadrants revealed distinct functional changes in high DEGs in S606 strains during 0–24 h of heat stress. In terms of metabolic pathways, high DEGs of S606 were mainly concentrated in amino acids and secondary metabolites after 6 h of heat stress. By 12 h of heat stress, DEGs in S606 strains were distributed in the tryptophan metabolism pathway, alcohol, and lactic acid metabolism, among which *ALDH* genes in the IAA synthesis pathway also showed high expression differences. This was consistent with the increase of auxin IAA content thereafter. In the signaling pathways, after 6 h of heat stress, the *Hog 1* gene in the hyperpermeability pathway and the *MKK 12* gene in the cell wall response pathway of S606 strain were highly expressed. By 12 h of heat stress, the *FUS 3* gene in the pheromone pathway, the WSC123 receptor in the cell wall response pathway, *Pbs 2*, *Hog 1*, *Gpd 1*, *Gre 2* in the hyperpermeable pathway, and various genes in the starvation pathway were highly expressed. After 24 h of heat stress, 14 genes in high-permeability pathways, cell wall response pathway, pheromone receptor pathway, and starvation mitogen-activated protein kinase (MAPK) signaling pathway, as well as DNA mismatch and recombinant repair, showed high differential expression. These results suggest that the heat-tolerant strain S606 improves heat tolerance by regulating secondary metabolic pathways and signaling pathways responding to stress as well as the expression of genes related to DNA repair. The simultaneous analysis of promoter regulatory elements in the upstream regions of signal regulation-related genes in S606 identified auxin response elements in the promoters of *WSC123*, *Ste 3*, *Cdc 24*, *Cla 4*, *Rom 1, 2*, *FUS 3*, *Pbs 2*, and *Hog 1*, and the cell growth cycle transcription factors *Tec 1*, *Ste 12*, *clb 1/2*, and *Gre 2*. This indicates that the S606 strain improved heat tolerance by increasing the expression of genes related to IAA content regulation and stress response and MAPK signaling response pathway at 24 h after heat stress (Fig. 7).

### Association analysis of the expression levels of transcriptome and hormone metabolites

To explore the effect of gene expression and the expression level of hormone metabolites on heat tolerance after heat stress, we performed OP2LS analysis. KEGG enrichment analysis of genes and metabolites obtained from OP2LS analysis revealed that genes related to metabolism during heat stress in S606 were significantly enriched in repair pathways, including cell cycle, cell autophagy, and homologous recombination (Fig. 6E through G). Notably, the expression of energy-related intermediate metabolite synthesis pathways, such as acetyl-CoA, increased significantly. These findings suggest that S606 improved heat tolerance during heat stress by enhancing cell cycle, autophagy, and homologous recombination as well as energy metabolism and other pathways. The results of the OP2LS analysis are highly consistent with those of the nine-quadrant analysis, which indicated that the S606 strain gained stronger heat tolerance by positively regulating the expression of stress signaling pathway, DNA repair, and energy metabolism-related genes. Furthermore, the increase in auxin IAA content supports the notion that S606 improved heat tolerance by modulating stress response and energy metabolism pathways. 

## DISCUSSION

As mentioned in the literature review, IAA synthesis in plants and bacteria is mainly metabolized through the Trp-dependent TAM, IPA, IAN, and IAM pathways. Several reports have shown that the yeast DMKU-CP293 strain produced IPA as the main pathway, and IAM and TAM as the complementary pathway (68). The current study found that *Rhizobium tropici* and *Fusarium* species synthesize IAA via the IPA, TAM, and IAM pathways. Additionally, the IAM pathway has been identified as a principal route for IAA synthesis in anthracis (*Colletotrichum gloeosporioides* [69], *C. acutatum* [70], and *C. fructicola* [71]). In *Magnaporthe oryzae*, IAA is mainly synthesized through the IPA pathway (72). Prior studies noted that fungal intracellular IAA biosynthesis predominantly proceeds through three Trp-dependent pathways, TAM, IPA, and IAM.

In this study, we assessed the levels of tryptophan along with metabolites associated with the TAM, IPA, and IAM pathways at various time points (0, 6, 12, 24 h) during heat stress at 40°C. What is surprising is that the concentrations of IAN and IAM pathways remained low and unchanged throughout this period. Despite identifying genes related to both the IAN and IAM pathways within mushroom genomes, metabolite detection revealed that these pathways were not efficient in synthesizing IAA. As far as we are aware, this is the first time in *L. edodes* strains that Trp-dependent synthesis of IAA primarily occurred via two key routes: TAM and IPA pathways.

Both previously published data alongside quantitative metabolomics analyses from this experiment demonstrated a significant increase in intracellular IAA content in *L. edodes* between 12 and 24 h post-heat stress exposure, indicating an enhancement in IAA biosynthesis under prolonged thermal conditions. Targeted metabolomics assessments measuring precursor levels for IAA synthesis in S606 strains subjected to heat stress at 40°C over intervals of 0, 6, 12, and 24 h revealed substantial alterations in metabolites linked to the IAA synthesis pathway under heat exposure. Notably among these changes was a marked increase in levels of Trp, TAM, and IAALD after 6 h of heat stress; conversely, the concentration of IPA exhibited a decline. The elevated IAA content was observed after 12 h of heat stress, with the timing of these changes significantly lagging behind that of anabolic intermediates. The most striking result is that the tryptophan content in the YS3357 strain is 175–240 times higher than that in S606. This abnormal accumulation of tryptophan may be attributed to its inability to synthesize other metabolites and its ineffective conversion into IAA. This observation may support the hypothesis that high intracellular concentration of TAM can exert a toxic effect on cells, which may represent a critical factor distinguishing YS3357 from S606. However, further experimental evidence is required to determine whether this is a common occurrence among heat-sensitive strains.

Tryptophan transferase facilitates the conversion of Trp into IPA, while *TDC* converts it into TAM, both are essential genes regulating IAA synthesis in various organisms. Previous studies have reported that the *TAA* gene regulates IPA synthesis in *Ustilago maydis* strain (55) and *Leptosphaeria maculans* (21) strain, the *Umtam1* and *tam2* deletion mutants (the tryptophan aminotransferase genes) exhibit the decreased IPA content, the exogenous addition of Trp rescues the IPA content decrease, and the expression of gene *LmTAM1* was upregulated after the addition of tryptophan, and its expression was positively correlated with IAA production. *Taphrina deformans* (73), *Metarhizium robertsii* (74), and *Cyanodermella asteris* (75) strains have been reported to possess *TDC* activity, and the exogenous addition of TAM in *Azospirillium*, *Ustilago maydis* (73), and *Leptosphaeria maculans* strains (21) enhances *TDC* activity, thus increasing the level of IAA. Overall, research on *TAA* and *TDC* genes within fungal species remains limited. In this study, we silenced the expression of both *LeTAA* and *LeTDC* genes in the *L. edodes* S606 strain. Our findings indicate that downregulation of these genes does not adversely affect normal hyphal growth but does reduce heat resistance, suggesting that the expression levels of *LeTAA* and *LeTDC* play a significant role in modulating hyphal thermotolerance. In this study, it was discovered that the heat-sensitive strain YS3357 accumulates a high concentration of TAM. Consequently, experiments involving the exogenous addition of tryptamine were conducted on both the heat-tolerant strain S606 and the heat-sensitive strain YS3357. Following heat shock at 40°C, the recovery phenotype of these strains was assessed. The results are depicted in Fig. 3. It appears that the exogenous addition of tryptamine at various concentrations does not influence mycelial growth. However, the application of high-concentration tryptamine does impact the recovery ability of *L. edodes* mycelia post-heat stress at 40°C. Further studies will compare the TAM degradation rate or transformation efficiency between YS3357 and S606.

Furthermore, fungal MAPK signaling pathway encompasses pheromone signaling, cell wall integrity signaling as well as responses to salt and hypertonic stress conditions. The fungal cell wall signaling cascade and hypertonic pathways typically respond to compensatory mechanisms associated with heat stress, resulting in the accumulation of intracellular trehalose (76–78). This process enhances intracellular osmotic pressure and leads to plasma membrane stretching (79–81). WSC receptors in yeast are capable of responding to heat stress and rapidly reacting to cell wall stress signals (82, 83); they also influence the hyphal growth and heat sensitivity (84). In this study, we performed a nine-quadrant analysis at 2, 4, 6, 12, and 24 h post-heat stress. The remarkable results revealed that most genes involved in the MAPK signal transduction pathway as well as those related to lactate, alcohol, and IAA synthesis in the *L. edodes* strain S606 were significantly expressed after 12–24 h of heat exposure. In contrast, these gene expression levels were either downregulated or remained unchanged in the heat-sensitive strain YS3357. Within the MAPK signal transduction pathway, we identified auxin response factor response elements within the promoter regions of 13 genes across various pathways (see attachment). It may be the case, therefore, that during the period of 12–24 h under heat stress conditions, auxin IAA positively regulates the MAPK signal transduction pathway to initiate the expression of stress-related genes (Table S7).

In conclusion, this paper investigates alterations in auxin synthesis pathways and related auxin synthesis metabolite changes under conditions of heat stress through transcriptomics approaches at different time points during thermal exposure. Our findings would seem to show that intracellular IAA synthesis primarily occurs via both TAM and IPA pathways in *L. edodes*; however, excessive TAM accumulation after heat stress contributes to decreased thermotolerance. Furthermore, increased levels of IAA are predominantly derived from IPA-synthesized IAALD. Gene silencing experiments targeting *LeTAA* and *LeTDC* within the auxin synthesis pathway demonstrated reduced thermotolerance in hyphae. Notably, elevated intracellular IAA content observed between 12 and 24 h during heat stress positively regulated genes implicated in the MAPK signal transduction pathway as a protective response against thermal damage. These results provide a deeper understanding of the mechanism of IAA synthesis in *L. edodes* strains, providing a molecular basis for future breeding of thermotolerant strains.

## Data Availability

The raw transcriptome sequencing data generated in this study have been deposited in the Genome Sequence Archive at the National Genomics Data Center (China National Center for Bioinformation) under BioProject accession number CRA016083. The data set are publicly accessible via https://ngdc.cncb.ac.cn/gsa/.

## SUPPLEMENTAL MATERIAL

Supplemental figures spectrum.01298-25-s0001.docx Fig. S1 to S5.

Supplemental tables spectrum.01298-25-s0002.xlsx Tables S1 to S7.

ASM does not own the copyrights to Supplemental Material that may be linked to, or accessed through, an article. The authors have granted ASM a non-exclusive, world-wide license to publish the Supplemental Material files. Please contact the corresponding author directly for reuse.
