# Supplementary material for: The alterations of the synthetic pathway and metabolic flux of auxin indole-3-acetic acid govern thermotolerance in Lentinula edodes mycelia subjected to heat stress
Source: Microbiol Spectr. 2025 Nov 28;14(1):e01298-25. doi: 10.1128/spectrum.01298-25 (PMC12772347; doi:10.1128/spectrum.01298-25)
Supplement: Supplemental figures — Fig. S1 to S5. [file spectrum.01298-25-s0001.docx]

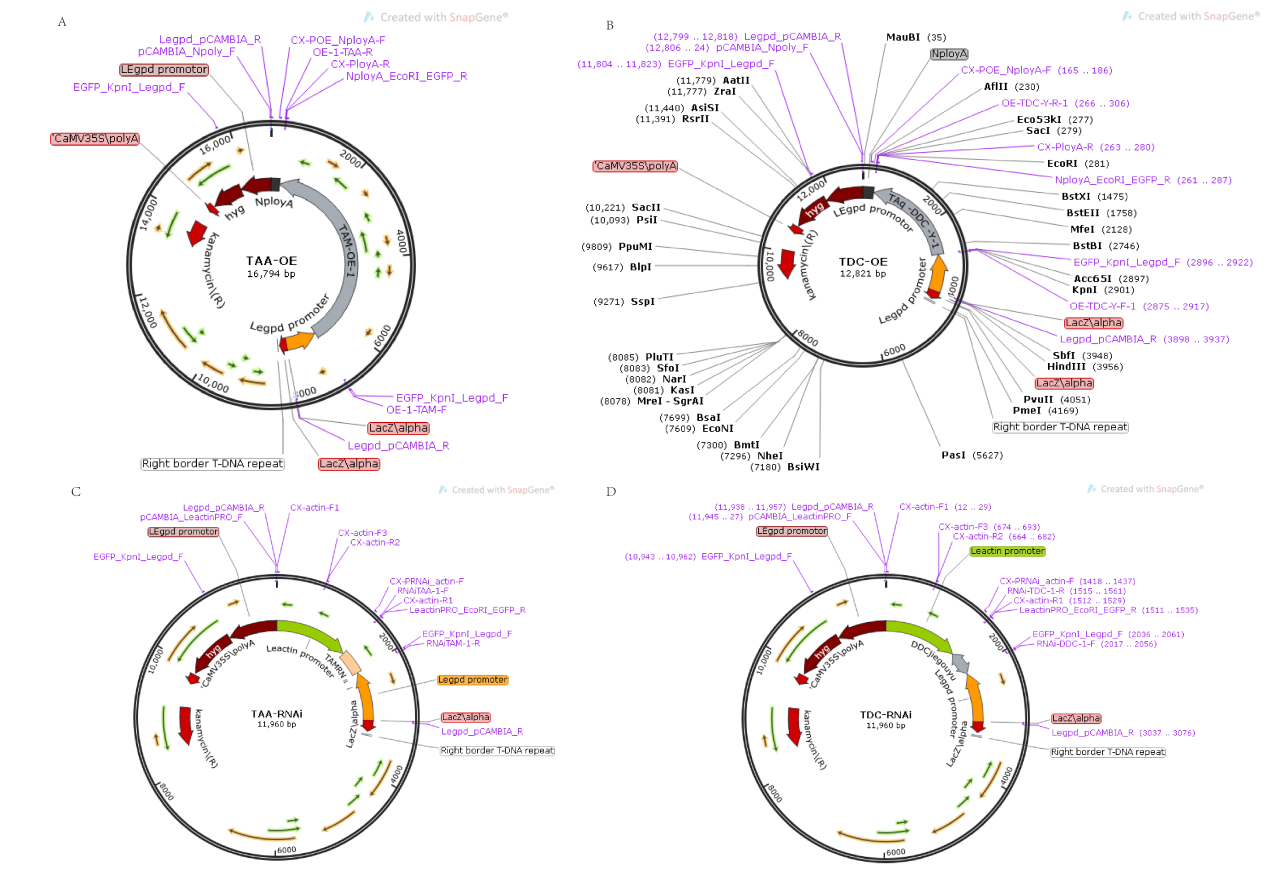


A

B

**Fig. S1.** Agrobacterium transformation vectors. **(A)** TAA-RNAi vector **(B)** TDC-RNAi vector.


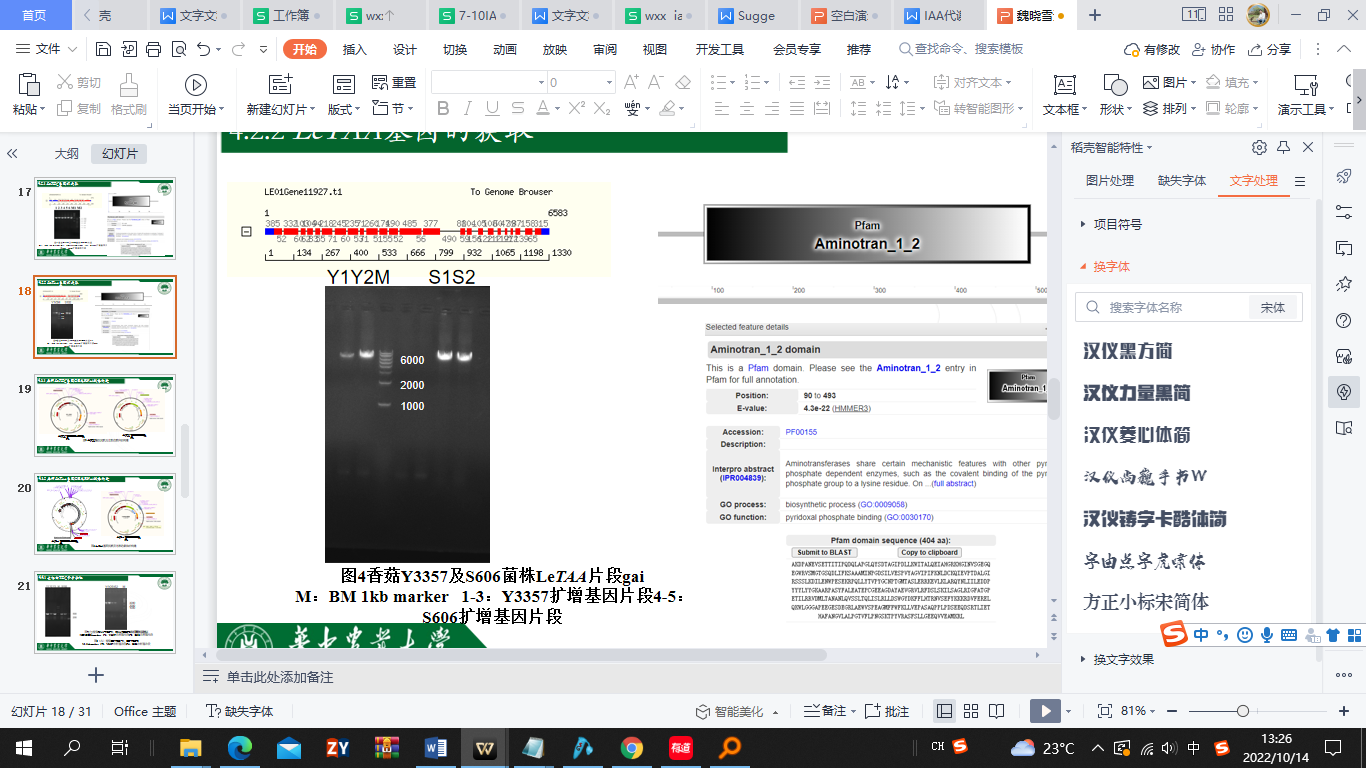


**Fig. S2.** LeTAA segments in YS3357 and S606 strains. **M**: BM 1kb DNA marker; **Lanes 1-2**: LeTAA amplification from YS3357; **Lanes 4-5**: LeTAA amplification from S606.


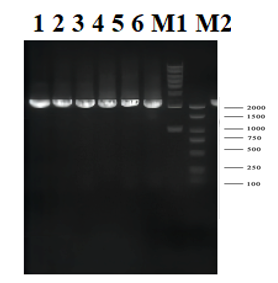


**Fig. S3.** LeTDC fragments in YS3357 and S606 strains. **M1**: BM 1kb DNA marker; **M2**: BM2000+ DNA marker; **Lanes 1-3**: LeTDC amplification from YS3357; **Lanes 4-6**: LeTDC amplification from S606.


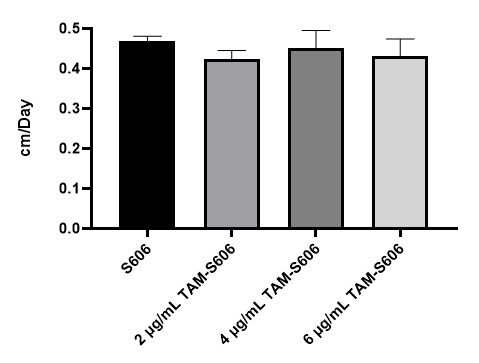

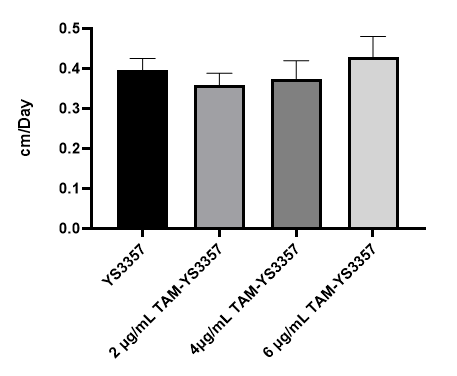


A

B

****Fig. S4.** Impact of exogenous tryptamine (TAM) on mycelial growth kinetics.**

(A) Heat-tolerant strain S606 growth rate under TAM supplementation.

(B) Heat-sensitive strain Y3357 growth rate under TAM supplementation.

Concentrations tested: 0 (DMSO control), 2000, 6000, and 12000 ng/g TAM. Error bars denote standard deviation(n≥3).


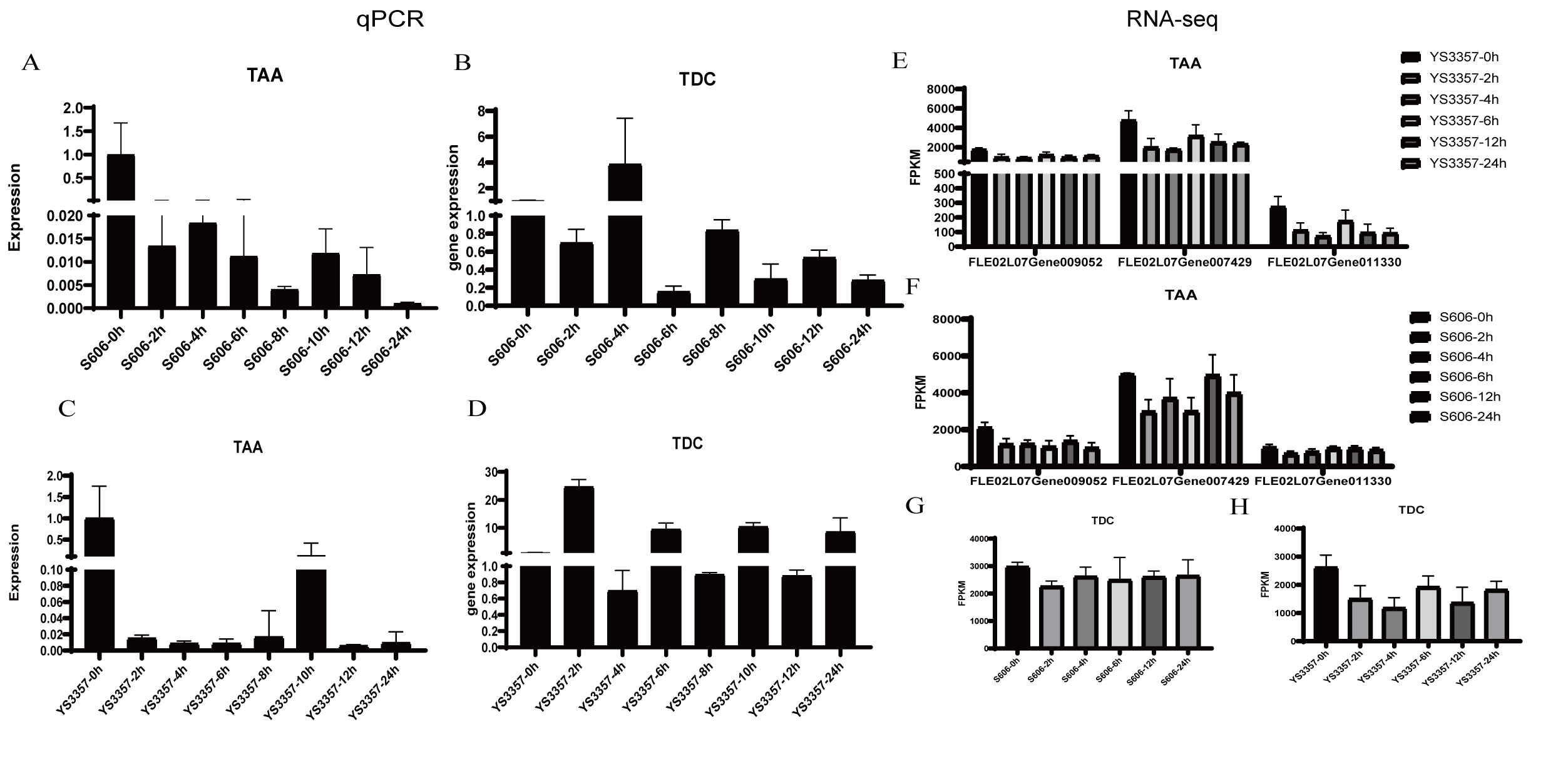


**Fig. S5. Expression analysis of *TAA* and *TDC* genes under heat stress.**

**(A，F) *TAA* expression in heat-tolerant strain S606 at indicated time points post-heat shock.**

**(B，G) *TDC* expression in heat-tolerant strain S606 at indicated time points post-heat shock.**

**(C，E) *TAA* expression in heat-sensitive strain YS3357 at indicated time points post-heat shock.**

**(D，H) *TDC* expression in heat-sensitive strain YS3357 at indicated time points post-heat shock.**

**Gene expression was quantified by qRT-PCR and normalized to [Reference Gene actin]. Data represent mean ± SD (n = 3 biological replicates). Heat shock conditions: [Temperature,40 °C].**
